# Exposing State Repression: Digital Discursive Contention by Chinese Protestors

**DOI:** 10.1007/s12116-024-09428-0

**Published:** 2024-08-13

**Authors:** Diana Fu, Christian Göbel

**Affiliations:** 1 Department of Political Science, Munk School of Global Affairs & Public Policy, University of Toronto, Toronto, Canada; 2 Department of East Asian Studies, University of Vienna, Vienna, Austria

**Keywords:** Repression, Contention, Protest, Citizenship, Social media, China

## Abstract

One of the major issues in international development is how disadvantaged populations mobilize in response to state repression. Whether in the Black Lives Movement or in the 2011 Arab Spring, digital exposures of police abuse have spurred social movements when people took to social media to expose it. Yet, in authoritarian regimes, citizens cannot easily initiate or participate in social movements. In such cases, how do victims of police violence express their dissatisfaction? This study examines this question in contemporary China, where repression of protesters is well documented. Based on a dataset of microblogs—Chinese tweets—documenting 74,415 protest events in the early Xi administration (2013–2016), this study analyzes how ordinary protestors, including migrant workers, peasants, and the urban poor, expose police abuse in social media. A close reading of microblogs documenting 150 randomly sampled events finds that Chinese protestors adopt three distinct narrative types: citizenship, solidarity, and confrontational. An accompanying quantitative analysis of the wider dataset further finds that ordinary protestors frequently expose police abuse online and that mentions of police abuse are closely associated with the above three narratives. Overall, this study contributes to understanding how abused protestors discursively contest authorities in the world’s most powerful authoritarian regime.

In the digital age, social movements around the world, from the Black Lives Movement to the Arab Spring, have shown how online platforms can amplify calls for social justice and expose state-sanctioned coercion. However, in authoritarian regimes like China where digital surveillance and censorship are pervasive, the dynamics of digital activism and social movements in the face of repression are much more limited (Yang 2020, Yang 2009. Lei 2018; deLisle et al. 2016). This study investigates how ordinary Chinese citizens, facing violations of basic social and development rights, navigate these constraints to voice their grievances online. The “authoritarian bargain” struck between many autocratic states and their citizens is such that the government must meet these basic development needs of its citizens in exchange for their acquiesce (Karshenas et al. 2014). When citizens perceive the government as failing to uphold these basic social rights, such as providing food, housing, healthcare, and employment, they may protest this injustice as a failure of the state, as was the case in the 2011 Arab Spring (Cammett and Salti 2018).^1^

In China, as elsewhere in the world, claims to food and healthcare are considered basic development obligations that the state provides for its population as part of the social contract between the state and society (Jurkovich 2020). Rulers have long justified the legitimacy of their rule on their ability to provide the basic subsistence and development needs of the people, prioritizing these before political rights (Perry 2008). Mao Zedong, founder of the Chinese Communist Party, emphasized rights not only to subsistence but also to development, which was defined as an improvement in livelihood for the population (ibid). Subsequent Chinese leaders, including Xi Jinping, have all emphasized socio-economic development as a bedrock of the party-state’s obligations to society (Solinger 2017). When the party-state fails to meet its obligations to provide basic development rights, ordinary citizens—peasants, migrant workers, middle-class homeowners, and others—sometimes take to the streets and/or social media to protest the broken social contract.^2^ Under normal circumstances, local authorities respond to protestors’ demands using a combination of repression and concessions (Cai 2010). However, when the police or other agents of coercion beat up or arrest protestors for claiming basic development rights, it adds fuel to the fire, thus igniting protestors to expose state malfeasance online.

This study analyzes protestors’ blogs to uncover how Chinese citizens narrate their experiences of repression under a powerful authoritarian regime that tightly controls online discourse. These narratives are about the state’s failure to meet basic development needs and its lack of response to street protests calling for remediation. Specifically, it analyzes the typology of digital narratives by everyday protestors, including peasants whose land has been grabbed, migrant workers in wage disputes, defrauded homeowners, and other ordinary citizens with largely nonpolitical grievances. Short of lighting a full-fledged social movement, their digital narratives are nevertheless consequential because, for one thing, online exposure of state abuse can lead to “unintended effects” downstream, including backlash mobilization (Hassan et al. 2022; Pan and Siegel 2020). For another, narratives can shed light on the less obvious motives of why people join movements, not necessarily because there is a clear-cut moral or rational impetus but because of the nonsensical nature of what is happening to them (Polletta 2006: 35). Importantly, these narratives can disrupt the state’s positive propaganda of its own image and governance, thus challenging state power. To the extent that the regime’s legitimacy depends upon controlling the public narrative about its power and performance, publicizing the abuse constitutes a form of discursive contention.

An analysis of the universe of blogs documenting 74,415 protest events in the “Wickedonna” dataset collected by two citizen journalists in China between 2013 and 2016 analyzes approximately 180,000 blogs of protest events. First, an analysis of the entire dataset finds that despite high levels of censorship, state repression is mentioned in nearly twenty-five percent of all protest events. This shows that even in an authoritarian environment with tight internet control, protestors do, in fact, regularly blog about official malfeasance online. Having established a baseline of blogs that mention repression, the study then probes the discursive content of blogs about abuse, with specific attention to the language they use—terminology, tonality, metaphors, and expletives—to compose digital narratives. A close reading of a random sample of 150 protest events (each documented by multiple microblogs) finds that Chinese protestors tend to adopt three distinct narrative types to talk about state repression: (a) a *citizenship narrative* that emphasizes their rightful claims to certain public goods or state protection, (b) a *mobilization narrative* that calls for collective action to mobilize against state repression, and (c) a *confrontational* narrative that expresses outrage or insults against power-holders. These are confirmed by a further quantitative analysis of the entire corpus of the protest events showing that mentions of repression are closely correlated with the adoption of the above typology.

## Repression and Mobilization Under Authoritarianism

This study contributes to existing literature on repression and mobilization in authoritarian settings, which can be divided into three fields of inquiry: (a) the relationship between repression and mobilization, (b) the factors that impact why states choose a certain strategy of repression over another, and (c) narratives of repression, to which our study contributes. The first field of inquiry is concerned with the relationship between repression and mobilization, termed the “repression-mobilization nexus” (Davenport 2005). Repression can have variable effects on mobilization (Carey 2010; Davenport 2005; Earl and Soule 2010). Under certain conditions, repression can decrease or curtail protests, thus achieving the state’s goals of demobilization (Jeffries 2002; Jones 1988; Oberschall 1973). Under other conditions, repressive actions such as tear-gassing and arresting protestors backfire on authorities by inciting more people to take to the streets (O’Brien and Deng 2015; Hess and Martin 2006; Goldstone and Tilly 2001; White 1989). In addition, the relationship can be curvilinear, meaning that repression can deter mobilization up to a point before it triggers the masses to revolt (Lichbach and Gurr 1981). Besides changing future contentious trajectories, repression can also have a long-lasting effect on changing political identities and cleavages (Blaydes 2018).

A second field of inquiry probes elites’ threat perceptions and why repressive agents choose certain types of measures over others. States have many repressive tools at their disposal; they may choose to apply direct coercion, such as torture, riot policing, and arrest (Davenport 2007), or instead opt for noncoercive instruments such as channeling (Robertson 2010, 179-80; Oberschall 1973). Moreover, repressive agents may choose to simply ignore protestors (Bishara 2015) or engage in strategic co-optation and infiltration (Lerner 2021; Mattingly 2020; Koesel 2014). Such choices may be conditioned by the repressive agent’s risk-and-benefit analysis (Goldstone and Tilly 2001), their perception of rights claims (Simmons 2021), and their capacity for distributing welfare in exchange for political support (Pan 2020; Mares and Young 2019). In liberal autocracies, elites’ threat perceptions may also be shaped by the degree of organizational capacity, with organized protests more likely to face police violence than spontaneous ones (Berman 2021).

A third, less populated, field of inquiry examines how repression is narrated by the population experiencing it. Narratives are closely associated with framing, which is the process through which contenders “assign meaning to and interpret relevant events and conditions in ways that are intended to mobilize potential adherents and constituents, to garner bystander support, and to demobilize antagonists” (Snow and Benford 1988, 198). The framing perspective in social-movement studies pays attention to how contenders and others interpret or assign meaning to the events that trigger mobilization (Snow 2007, 384). Frames are strategically crafted by challengers who wish to motivate others to take collective action. Not all frames are readily available, and social-movement entrepreneurs need to capitalize on pre-existing symbols, myths, and identities that can be appropriated for framing work (Simmons 2016, Ch 3). Narratives can also take many forms and purposes, such as expressing the confusion of experiences without a call for action that is part of frames (Polletta 2006, 45).

Political scientists in the qualitative tradition have long treated oral history as a valuable source of data (e.g., Wood 2003; Patterson and Monroe 1998). Yet, there are relatively few studies that document and analyze narratives of state repression. Among them, Kurzman’s (1996) study of the Iranian Revolution highlights the importance of studying protestors’ perceptions of the state’s coercive power (161). Wedeen’s detailed ethnography of the lives of repressed Syrians under Assad shows that citizens do not talk openly about repression but rather act as if the Assad cult and all its accompanying paraphernalia were real (Wedeen 1999). Pearlman’s documentation of the narratives of Syrian refugees reveals evidence of four different types of fear: silenced, surmounted, semi-normalized, and nebulous (Pearlman 2016, 24). Moss’ interviews with Libyan and Syrian exiled activists reveal narratives of transnational repression that show continued fear of speaking out against the regime, even while residing abroad (Moss 2016). In addition, the circulation of rumors under the Ba’athist regime in Iraq represents another form of narrative of repression (Blaydes 2018, 196-7), except for those rumors that circulated privately, in contrast with the public blogs in this study that are public. Narratives of repression can also have an impact on political behavior. Citizens may tell each other “control parables,” didactic stories about previous repression, that serve as warnings to others, hence inducing a form of self-censorship (Stern and Hassid 2012). Narratives, when crafted with the intent to tell a story that motivates people to join a social movement or a contentious action, can be the basis of collective action frames. They can also be used as a tool of state control, such as when the government compels dissidents to narrate their crimes and repentance via public confessions, thus displaying an “indoctrination dimension of repression” (Fu 2023). Together, these studies draw attention to when and how citizens in illiberal settings have narrated their experiences with repressive state abuse through a range of responses from silence to defiance.

This study extends this third field of inquiry about narratives of repression by examining public digital narratives of abuse by coercive agents under arguably one of the most repressive administrations in recent Chinese history—the Xi Jinping administration (2013–present). Being attuned to the broader range of narrative types that everyday protestors articulate sheds light on the different ways that protestors digitally contend with the state. These narratives are a valuable source of interpretive data on citizens’ self-perceptions of power related to the state and their discursive agency in contesting official narratives.

## Data and Methods

This study draws upon a dataset of approximately 180,000 microblogs or “tweets” documenting 74,415 protest events staged by ordinary citizens across China between January 2013 and June 2016.^3^ The unit of analysis for quantitative components of the study is the protest event, which encompasses all microblogs documenting a single occurrence of protest.^4^ Each protest event—defined as a contentious gathering where participants openly express a grievance or make claims against state or private actors—is documented on average by 2.5 microblogs.^5^ This dataset, alternatively known as Wickedonna or “Not the News” (*fei xinwen*), was collected and verified by two Chinese citizen journalists before the government could censor them. They provide the most complete coverage of Chinese protest events in the period of observation in which the number of protests was still increasing, suggesting that the dataset captures a period of high protest activity during Xi Jinping’s first administration (Chen 2020).

The individuals whose microblogs we examine in this study are everyday protestors with everyday grievances about livelihood issues. As Appendix 4 Table 4 shows, protest issues related to labor and real estate are especially prevalent. These protesters differ from “digital radicals” (Yang 2020) who use social media to raise awareness and call for action on specific advocacy issues. They also differ from online opinion leaders who have sizeable social media followings and are explicitly attempting to influence public opinion on social issues. The microblogs of ordinary protesters are a valuable source for understanding the ways in which normal citizens in China express their encounters with authorities and the diverse ways in which their discourse disrupts officially sanctioned narratives.

To determine how often protestors blogged about repression, the team identified all the events in the dataset that contained keywords mentioning state repression, defined narrowly as being “beaten up” (*ouda*) and/or “arrested” (*daizou*).^6^ Using a word2vec word-embedding model trained on the Wickedonna dataset, the team identified synonyms of these terms, one appearance of which qualifies as a mention of repression (see Appendix 1). Next, the team sought to analyze how people narrate experiences of state repression. To do this, we randomly sampled 150 protest events to do a close reading of the terms, as well as the tonality, content, and context of the microblogs collected for each event. From this close reading, we constructed a typology of narratives of repression that reveal how individuals come to make sense of their encounters with authorities.

Finally, the team tested if the typology of narratives from our qualitative study is systematically related to mentions of repression and is thus generalizable to the broader population of protest events. In other words, is there a statistical association between a mention of state repression and the adoption of one or more of the above narratives in the microblogs documenting 74,415 protest events at large? Following the procedure for coding repression, we also classified all protest events by the presence or absence of terms associated with at least one of the three narratives: citizenship, mobilization, and confrontational. To determine the presence of each narrative, the team first derived key terms associated with each of the three narrative types through a close reading of several hundred events.^7^ The team then trained a word2vec model on the dataset and used it to assemble dictionaries of synonyms. These synonyms are characterized by a small cosine distance from the key terms (see Appendix 2 Table 2).^8^

There are several limitations to this mixed methods study. One is that the data captures a span of time marking the early Xi Jinping era (2013–2016), a period of transition from the Hu-Wen era (2002–2012) which was relatively more liberal. However, protests and strikes were still quite frequent across China in this study’s period of observation compared to more recent years (Zhang and Pan 2019; Chen 2020), which may mean the data on protest blogging is not representative of the second Xi era (2017–2022). Second, the data in this study does not tell us why certain protestors blogged about their experiences while others did not. Thirdly, the data does not allow us to assess the baseline percentage of protestors who experienced repression but chose not to blog about it. Finally, we could only access the blogs as a form of digital narratives, which are by nature more clipped than oral histories. For ethical reasons and because it was often not possible to trace who wrote these blogs, interview data was not collected.

## Chinese Protestors Routinely Blogged About Repression (2013–2016)

**Fig. 1:**
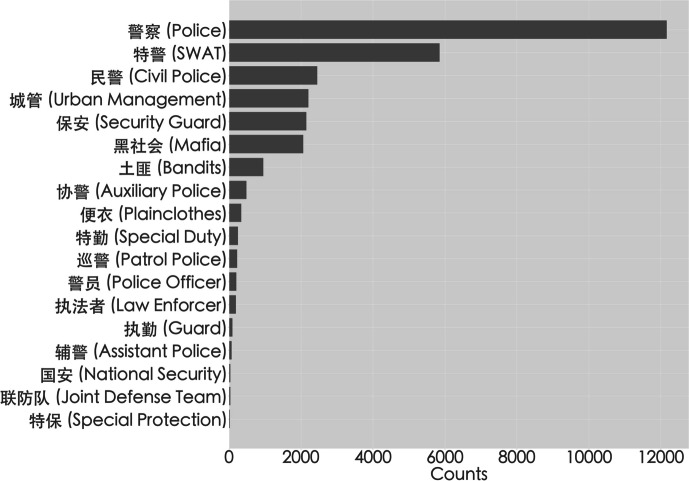
Frequency mentions of agents of coercion

China is a high-capacity authoritarian state that has increased both post-facto and pre-emptive repression, making it risky for citizens to protest or demonstrate (Greitens 2020; Truex 2018). Under Xi’s leadership, the party-state has invested in a formidable coercive apparatus, the primary purposes of which is to ensure social stability (Scoggins and O’Brien 2016; Wang and Minzner 2015). This apparatus has also incorporated nonstate actors such as the mafia (Ong 2018, Chen 2017), civil society groups (Mattingly 2020), and familial networks into its repression apparatus (O’Brien and Deng 2017). In the early years of the Xi administration (2013–2015), the coercive capacity of the state, measured by provincial public security expenditure per capita, was positively correlated with overt repression in the form of violence and arrests by the police and thugs (Li and Elfstrom 2021). China’s under-resourced frontline police often have “everyday violence” encounters with society as they mishandle crimes, which then feeds back into a loop of social unrest (Scoggins 2023, Ch. 6). Under the Xi administration, police have been more willing to arrest protestors, as well as use surveillance and repression to crush them (Chen 2020). In particular, the administration has placed more emphasis on reforming “high policing activities” such as protest control and surveillance over “low policing activities” such as everyday crime control and traffic violations (Scoggins 2023). In this respect, the party-state has shown a vested interest in a public image of the People’s Armed Police (PAP) as both a responsive and a restrained force, one that showcases the police working hard to improve society (Ibid: 266). In addition to the police, a paramilitary force responsible for riot control since 2018, there is also the auxiliary police (*xiejing* or *fujing*), urban management officers (*chengguan*), thugs hired by the authorities (*hei shehui*), and security guards (*baoan*), among others (Scoggins 2023: Ch1; Ong 2022; Chen 2017). However, by far, the most referred to agent of repression by protest bloggers was the police (see Fig. 1).

In the context of clashes between these agents of coercion and society, peasants, migrant workers, middle-class residents, and other ordinary citizens have continued to press for basic development rights during Xi Jinping’s first administration (2013–2018). These included labor rights, property rights (real estate disputes and land grabs), protection from fraud, rights to adequate transportation, education, clean air, among others (see Göbel 2019, Figure 2.6). Few of these rights claims were about changing public policy or overthrowing the regime, as protestors understood that leveraging existing policies promising social welfare is much more likely to be tolerated by the government than larger-scale agitation for broad political change. When local authorities arrested or beat up protestors, some victims took to social media to expose police malfeasance in the hopes of redress, attracting media attention, or starting an online social movement.

Contemporary China is a place where it is highly risky for citizens to expose state-sanctioned coercion publicly. As early as the mid-1990s, the Chinese government was already controlling netizens’ access to content via censorship, law, and regulations (Yang 2009). Since the beginning of the Xi Jinping administration in 2013, Chinese citizens can be imprisoned for up to three years for defamation if false information posted on the internet is reposted more than five hundred times or viewed more than five thousand times.^9^ A 2015 amendment to the law criminalizes “false information regarding dangerous situations, the spread of diseases, disasters and police information, and those who seriously disturb social order,” which can carry a sentence of as many as seven years in prison. Finally, spreading “rumors” can also lead to a charge of “picking quarrels and provoking trouble,” with a maximum prison sentence of five years. In 2018, the government launched a mobile app that allows the public to report online rumors and uses artificial intelligence to detect content that disrupts social stability.^10^ In addition, the authorities jailed several influential Big V bloggers whose large followings on social media generated the potential for undesirable collective action.^11^

Despite the potential risks to publicly critiquing the party-state as described in the previous section, the quantitative analysis of the entire dataset with approximately 180,000 microblogs found that Chinese protestors do, in fact, blog about repression and do so routinely. Nearly 25% of all the protest events (each with one or more microblogs) in the corpus contained at least one keyword signaling repression (i.e., synonyms of being beaten up or arrested) (Appendix 1). This suggests that despite the high risks, Chinese protestors posted about personal experiences of repression quite frequently. Some of the microblogs may have been censored after they were captured by Wickedonna, but the findings nevertheless show that a sizeable number of protestors speak out online about being beaten or arrested by authorities, despite the risks.

There may be several reasons as to why protestors might choose to strategically blog about being man-handled by the authorities or attacked by private security agents despite the risks. For one, they may be participating in a type of “rightful resistance” (O'Brien and Li 2006) that Chinese rural protestors have long engaged in. Such resistance tries to catch upper-level officials’ attention by exposing lower-level authorities’ abuse of power. For another, blogging about grievances and abuses may generate publicity for the protestors’ causes. Even in a country where officials are unelected, negative publicity can incentivize government authorities to be accountable to the promises that they have made to citizens (Distelhorst 2017). Moreover, protestors may also be incentivized to blog about their sufferings at the hands of authorities in the hopes of rallying the crowd to take collective action, thus pressuring authorities to correct their wrongs. Such online collective action has, in the past, occasionally been successful at pressuring the state to make policy changes, as evidenced by the Sun Zhigang incident in 2003.^12^

The fact that digital contention of this nature continues to unfold even under repression and censorship is in keeping with the emergence of a broader “contentious public sphere” (Lei 2018) and a feedback loop in which dissatisfaction with everyday policing fuels further social unrest (Scoggins 2021). Even in the early years of the internet in the 1990s, Chinese netizens were still able to engage in “everyday forms of resistance” (Yang 2009, 55) that circumvented restrictions. Since 2005, the contentious public sphere in China has become nationwide, with a growing number of “public opinion incidents”—online discussions around a viral topic—threatening the party-state’s efforts to maintain social stability online and offline (Lei 2018). Chinese microblogging platforms enabled bloggers to actively disseminate commentary on public affairs, with accounts on the largest, Sina Weibo drawing tens of millions of online followers.^13^ Survey and experimental evidence in China show that individuals who are aware of being censored end up posting more about the banned topic as well as complaining about government censorship (Roberts 2018, 121–146). Digital critics have adopted several creative rhetorical tactics such as developing cyber vocabularies, using jokes and parables to poke at authorities (Han 2018, 88). Moreover, multiple “public opinion incidents”—online exposure of an event or an issue that then goes viral—can ultimately undermine faith in the regime (Scoggins 2021). In this context, the fact that ordinary Chinese protestors routinely blog about state-sanctioned coercion is not unexpected.

### Blogs Disrupt Regime Narratives

However, such online exposures of state-sanctioned coercion are important because they disrupt the regime’s “public transcripts” (Scott 1990) about its own image and legitimacy. Authoritarian states seek to enhance state power by constructing narratives that disguise its unsavory elements by staging rituals such as marches, parades, and officiating at ceremonies that require citizens to participate in the symbolic dominance of the state (ibid). In the digital sphere, such governance rituals include positive propaganda about all aspects of governance and rulers. In fact, the Chinese state under Xi Jinping has invested enormously in projecting a positive image of itself through both soft and hard propaganda (Huang 2015). It does so through a combination of censorship and “public opinion or sentiment guidance.”^14^ Under the Xi Jinping administration (2013–present), the regime has sought to minimize the “negative energy” online (e.g., posts about state abuse) and to maximize “positive energy” by circulating narratives that make the party-state appear as a benevolent and responsive power (Chen 2022: 40; Brady 2017). In 2019, the Cyberspace Administration of China instructed online content creators to “cultivate a positive and healthy network culture.”^15^ A key aspect of this guidance is to go beyond “strategic censorship” (Lorentzen 2014) by diverting and redirecting public opinion.^16^ The Chinese Communist Party has recruited an army of online users commonly referred to as “the 50 cent party” to strategically distract netizens by “cheerleading for the state…” (King et al. 2017: 497).

By articulating experiences of repression online, protestors disrupt the state’s important political work of embellishing its power through public opinion guidance—attempts to steer public opinion through censorship and by seeding the internet with pro-regime messages. Even as censurers race to scrub digital diaries of discontent from social media, these blogs nevertheless serve their disruptive purpose by explicitly exposing coercion suffered at the hands of the police or other agents. This pierces the imagery that the party-state seeks to project of itself—one of a benevolent and law-abiding regime (Gallagher 2017; Wang 2014; Perry 2008).

## Typology of Digital Repression Narratives

What characterizes digital narratives of state repression in contemporary China? A close reading of 150 randomly sampled events in the database finds that protestors construct three types of narratives in response to experiences of repression: a *citizenship* narrative, a *mobilization* narrative, and a *confrontational* narrative (see Table 1). All three types reflect protest bloggers’ interpretations of their relationship to authorities’ discourses. Table 1 shows that they see and speak of themselves as rights-deserving citizens, who attempt to mobilize and verbally confront authorities.

**Table 1:** Typology of digital repression narratives*

Narrative type	Definition	Associated terms
Citizenship	Expressions of entitlement with language of rights and rule of law; assumes authorities are beholden to providing basic social and subsistence rights	Rights protection (*weiquan*), law (*falü*), urban resident (*shimin*), rights (*quanyi or quanli*), or citizen (*gongmin*)
Mobilization	Expressions of grievances as a collective problem; calls for collective action	Help forward (*bangmang zhuan*), help spread (*bangmang zhuanfa*), save us (*jiu women*), calling on everyone (*qiu dajia*), and help us (*bang women*)
Confrontational	Expressions of anger directed at authorities; use of profanity attributing blame to authorities	Angry (*fennu*), bandits (*tufei*), popular anger (*minfen*), and damn it (*tamade*)

*See Appendix 2 Table 2 for a full glossary of terms associated with each narrative and endnote 22 for an explanation of the context of the lexicon

### Citizenship Narrative

A citizenship narrative type refers to the articulation of one’s positionality and claims with the language of rights and the rule of law (Perry 2008; O'Brien and Li 2006). It contrasts with a subject narrative in which the writer positions themselves in a supplicant position, begging for rights from authorities (Distelhorst and Fu 2019). The term “citizen” (*gongmin*) literally translates into “public person” and connotes a Confucian notion of public service (Goldman and Perry 2002: 4). In 1953, the newly established People’s Republic of China substituted “nationals” (*guomin*) with “citizen” (*gongmin*), enshrining it in the national constitution. Nevertheless, the political discourse of the Chinese Communist Party has long employed “the people” (*renmin*) to demarcate the difference between the masses who were obedient to the Party and political enemies (Ibid). In the post-1989 Tiananmen Democracy Movement period, “citizen” became adopted by dissidents and activists, some of whom spearheaded the “New Citizens’ Movement”^17^ to champion the rights of ordinary people (Pils 2015). The embrace of this term by those whom the regime deemed political challengers imbued it with a much more subversive connotation.

In addition to subversive connotations, citizenship narratives can also include constructive suggestions about public policy. A recent study examining the rhetoric that people employ when speaking to officials online finds that they choose texts that convey a type of “constructive citizenship” in which the citizens offered governance solutions to state officials via an institution called mayors’ mailboxes in China (Brown 2021a). Such solution-oriented rhetoric is characterized by a positive relationship with authorities, civic consciousness, and political efficacy or rational persuasion of officials (Brown 2021b).

This study’s definition of citizenship narrative follows in the tradition of “rightful resistance” (O'Brien and Li 2006) which hinges references on laws and obligations that the state has passed but has not fulfilled. Specifically, a citizenship narrative assumes that the authorities are beholden to providing the bundle of rights that is promised by the law. While the Chinese party-state has a narrow conception of the law as a technical institution, Chinese netizens conceive the law as being intimately related to citizens’ rights, human rights, and the law’s moral qualities (Lei and Zhou 2016). Reference to the law is therefore an integral part of the citizenship identity, and netizens who identify as citizens are motivated to contest official discourses online and help others (Lei 2018: Ch.6). Moreover, the law is often mentioned in relation to claiming social rights, which are a bundle of subsistence rights including basic food, housing, economic development, and protection.

A close reading of the microblogs documenting 150 protest events illustrates that those mentioning repression sometimes contain a citizenship narrative. In other words, individual protestors or observers who blog about their encounters with repressive authorities also tend to speak of themselves as citizens with rightful and legal claims. In these cases, repression emboldens people to demand their rights on an equal footing with the authorities rather than as supplicants. The keywords that were coded as articulating a citizenship narrative included terms such as “rights protection” (*weiquan*), law (*falü*), “urban resident” (*shimin*), “rights” (*quanyi or quanli*), or “citizen” (*gongmin*) (see Appendix 2 Table 2).

The citizenship narrative is illustrated by a microblog such as this one of a 2016 incident in Shaanxi province in which protestors took to the streets demanding a government response to the forced demolition of their homes. The blogger calls attention to the protestors’ struggle against the local state which hires thugs to repress them.^18^ The microblog mentions both the citizens’ claims to a livelihood and the local authorities’ hiring of thugs to demolish homes:

They have petitioned multiple times about the municipal government’s hiring of thugs to forcibly demolish [their homes]. Today, they were forced to use this extreme method to express their claims: we want our *livelihoods*, we want to live!^19^

The microblog was accompanied by photos of protestors holding a banner that read, “Resist violent demolition, protect *citizens*’ lawful rights [emphasis added].” In this case, the banner directly denounces coercion and urges people to protect “citizens’ lawful rights.” The use of “citizen” (*gongmin*) here emphasizes that the protestors feel entitled to claim their rights free of harassment. Its emphasis on livelihood accords with an interpretation that the government is breaking its promises of guaranteeing social rights, which has been the foundation of regime legitimacy since the imperial era (Perry 2008). It contrasts with a subject discourse in which protestors beg authorities to intercede on their behalf.

Another microblog complaining of violence involved a demonstration against the large-scale embezzlement of land-sales revenue by local officials. Adopting a citizenship narrative, this blog referenced their legal rights:

“We don’t want to resort to brute force; *we just want to protect our own rights*. In the struggle between the public and the officials, the side that suffers a bitter defeat is clearly evident […] We protest to *assert our rights* and will continue to do so until we get an explanation. If the issue becomes widely known, who will have the last laugh? I believe it will certainly be the side with *justice* on their side!”

Notably, this blogger expressed that the authorities were acting against the law by allegedly misappropriating public funds from land sales, suppressing the villagers’ lawful protests, and denying their rights to seek legal redress. This violates a basic promise of protection, which is integral to the guarantee of social and development rights (Perry 2008). There is no hint of a subject narrative, only defiance at what the blogger perceived to be unjustifiable state repression.

Other bloggers also questioned the legality of authorities’ actions by adopting a citizenship narrative that emphasized the law. For instance, in decrying police brutality, villagers in Guangdong province questioned the lawlessness of the police:

We petitioned *according to the law* but did not receive the support of the government. The village officials forcefully took away all of the documents and beat the villagers. Just when we thought that Xi Jinping would bring us *justice*, the darkest of village governments united with local police to bully us…*where has the law gone*? [emphasis added]^20^

This blogger reported that when the villagers sought to pursue legal means—petitioning—to advocate for their rights, they were ignored. The local authorities and the police colluded in confiscating documents and beating the villagers. Notably, the microblog follows this description of repression with claims for justice and the rule of law. In posing the rhetorical question of where the law has gone, the blogger is speaking not as a supplicant but as a citizen who expects the government to act according to the constraints of the law.

The adoption of a citizenship narrative by protestors directly contended with the Chinese party-state’s propaganda on its commitment to guaranteeing social rights and on rule-by-law governance that served the people. Between 2013 and 2016, the Xi administration continued to extend and revamp the “minimum livelihood guarantee” programs which is an urban social assistance program aimed at reducing poverty (Solinger 2017). At the same time, Xi’s signature anti-corruption campaign was under way, which also urged officials to “carry the sword of justice and scale of equality” while cracking down on nonresponsive police or power abuse.^21^ By tapping into this rule-by-law discourse, protest bloggers exposed that, in fact, the government was not keeping its promise of basic protections and was thus behaving like an illegitimate power. They used a citizenship narrative to discursively contend with the state’s own narrative about being a responsible power that guaranteed basic rights to subsistence and protection.

### Mobilization Narrative

In addition to adopting a citizenship narrative, protestors who blog about experiences of repression also deployed a mobilization narrative, which makes an appeal to netizens to either join in their collective action or to recirculate their post. Such narratives are similar to collective action frames that “activate adherents, transform bystanders into supporters, exact concessions from targets, and demobilize antagonists” (Snow 2007: 385). By reappropriating the discourse of collective action employed by the party-state, the protestors discursively contended with the regime, defying the latter’s monopoly on mobilizing citizens in state-led campaigns. Specifically, abused protestors attempt to mobilize other netizens to help them popularize their grievance by using words that call for their posts to be amplified via recirculation such as “help forward” (*bangmang zhuan*), “help spread” (*bangmang zhuanfa*), “save us” (*jiu women*), “calling on everyone to…” (*qiu dajia*), and “help us” (*bang women*) (see Appendix 2 Table 2).^22^ It is common for protest bloggers who viscerally critique the local government’s actions to also call on observers to circulate their microblogs in the hopes of generating publicity. This could pressure officials to deliver their promises in what has been termed “publicity-driven accountability” (Distelhorst 2017). For example, in 2015 a group of protestors in Shanghai demonstrated in the streets for seven consecutive days against the local government’s proposal to build a chemical plant near a residential district. In their microblogs, they first narrated the repression they faced:

June 28^th^ is the seventh consecutive day that the people of Jinshan have taken to the streets to protest! We cannot hide our angry sentiments! If we had not been pressed to the point of desperation, we would not have exploded! Today, the police and coercive forces have started to target us physically… (Blogger A).^23^

Having described being targeted by the police, bloggers also called for the circulation of their microblogs which sought to mobilize collective action against the authorities:

We are not rioting; we are not wreaking havoc! We just want to express our thoughts! *Begging for circulation widely* [emphasis added] … You people feed off of taxpayers’ money, does that mean you can bully commoners?... *Begging everyone to circulate rapidly, circulate the world three times, let the whole world see*! (Blogger B).^24^

The blogger’s appeal for recirculation of the microblog was echoed by many others in the database. By calling for circulation, protestors were hoping that other netizens would provide mobilizational support for their cause.

Still other microblogs go beyond calling for circulation to directly mobilize collective action that defies the circumscribed boundaries set by the party-state. For example, the following post is about a demonstration to protest a tragic incident in which a local villager was allegedly beaten to death by police officers during a gambling raid. According to eyewitness accounts, the man was sitting under a tree near the gambling site when he was confronted by the police. In his attempt to escape, the post claims, he was chased by three officers into a mud-brick house with no exit. There, he allegedly was handcuffed and brutally beaten, leading to his death. The incident sparked outrage and grief, particularly among the victim’s family and the local community that took to the streets in protest. In November 2013, bloggers posted information and footage of the event and attempted to mobilize netizens:

[A] villager was *brutally beaten to death by three police officers* [emphasis added] [...] because of gambling, and all his belongings were taken away, including money, mobile phone, and rings. He was handcuffed, and the three police officers took turns beating him. *Everyone, please help spread this* to return justice to the deceased [emphasis added]*.* Is it just for gambling that someone should be beaten to death? Is it right for a people’s police officer to oppress the common people like this? I am speechless…^25^

This shows that despite the political risks, ordinary protest bloggers continued to mobilize netizens in the hope that their posts would go viral and generate broad attention. Recent research in China shows that viral posts that either call for collective action or vitriolically criticize top leaders do, in fact, trigger media coverage (Gallagher and Miller 2021). Regardless of whether protestors’ microblogs go viral, the adoption of such mobilization narratives and the experience of being beaten and/or arrested can themselves challenge the official references to solidarity and collective action, which are deployed for the purposes of state-led movements such as disaster relief or uniting against a common foreign enemy. Hence, by calling for solidarity outside of the context of state-mobilized movements (Ekiert and Perry 2020), the protest bloggers are discursively contesting the party-state.

### Confrontational Narrative

Finally, protestors blogging about repressive experiences also adopt a confrontational narrative, with expressions of anger that insult or attribute blame to the authorities. Unlike the citizenship and mobilization discourses, confrontational narratives are often nonstrategic and vent against authorities. Confrontation is commonly expressed through angry rhetoric such as “angry” (*fennu*), “bandits” (*tufei*), “the peoples’ anger” (*minfen*), and “damn it” (*tamade*) (see Appendix 2 Table 2). Sarcastic anger is expressed by rhetorical questions such as “Aren’t the police supposed to protect us?” and “so this is how much a life is worth?” or statements such as “turns out somebody has to die before you get any attention.”

Such verbal confrontation is itself a type of digital contention because it disrupts the state’s efforts to control emotional expressions online. Under the Chinese government’s rule, emotional management is central to state control, which entails the party’s manipulation and exploitation of the crowd’s emotions for mobilizational purposes (Perry 2002). Continuing this governance tradition, the Xi administration launched a concerted campaign to “civilize the web” by encouraging expressions of positive emotions while suppressing that of negative ones online (Yang 2018). When bloggers publicly display anger at the government for having repressed them, their words constitute a type of digital contention against the state, disrupting its concerted campaigns to manage emotional expressions.

A careful reading of microblogs in the random sample of 150 protest events showed evidence of unbridled anger publicly expressed toward abusive authorities. For example, in 2013 in the Eastern province of Anhui, street vendors protested the coercive behavior of urban management officers:

Hefei’s urban management officers have started to act up again. You guys get paid by taxpayers’ money, but you act like lords. This justice is *bullshit*! [emphasis added] A mass incident broke out on the pedestrian-only street of Hefei, with venders protesting against the unlawful behavior of urban management officers. The surrounding officers all got mobilized, beat up the commoners. This is *outrageous* [emphasis added] to both people and the gods; these officers are terrible!^26^

The use of profanity reveals the anger that this blogger has toward local authorities who have beaten up street vendors to disperse their collective action. The blogger does not use any guarded language to disguise their outrage. Instead, they confront the urban management officers directly, without couching their emotions in any legal language or even appeals to superior officials.

In other cases, the protest bloggers directly confront the local government, as evidenced in a microblog about proprietors’ protests in 2014 in Henan. The group of protesting urban proprietors had invested in a property that was severely delayed in its construction, and they had purportedly petitioned the government for eight years to no avail:

Government inaction. Proprietors have petitioned for eight years with no result…Before Christmas, proprietors self-organized to collectively demand our money back or for construction work to begin. Between noon and evening, several people were secretly taken away by the local police, no idea where they are! *Are they* [the local police] *or the mafia*?! [emphasis added].

This blogger’s anger was manifest in the decrying of police repression toward the proprietors. The sarcastic question “Are they police or the mafia?” suggested that the blogger is outraged by the behavior of the local government who, they sarcastically suggested, had hired the mafia to repress them.

Likewise, a 2015 blog about the protests over land grabs in the southern province of Fujian used sarcastic language to interrogate the government’s commitment to serving the people:

Because the head of the household refused to move, they [the authorities] used fire extinguishers on the elderly and beat them. Why is our Chinese government so corrupt? Why is it that government officials act so recklessly? Is it because they have somebody supporting them behind the scenes? There are too many questions…*on the surface, they say they’re serving the people, which sounds good…but dammit,* what has society nowadays become? [emphasis added]. 27

This microblog critiqued the authorities’ forced land-grab behavior and repression of those who resisted. The blogger denounced the government for paying lip service to “serving the people,” a key slogan of the Chinese Communist Party rule.

Together, these critical and often seething narratives challenged the state’s attempts to restrain the masses from expressing negative emotions online in an effort to manufacture a semblance of “positive energy” (*zheng nengliang*). When protest bloggers write extensive texts about their experience of being beaten up or detained by the authorities, they disrupt the regime’s “public transcripts” (Scott 1990) about the nature of its power. While each of the narratives has a varying degree of threat to the regime, all three detract from the party-state’s euphemistic portrayal of itself. Such disruption lies at the heart of discursive contention.

**Fig. 2:**
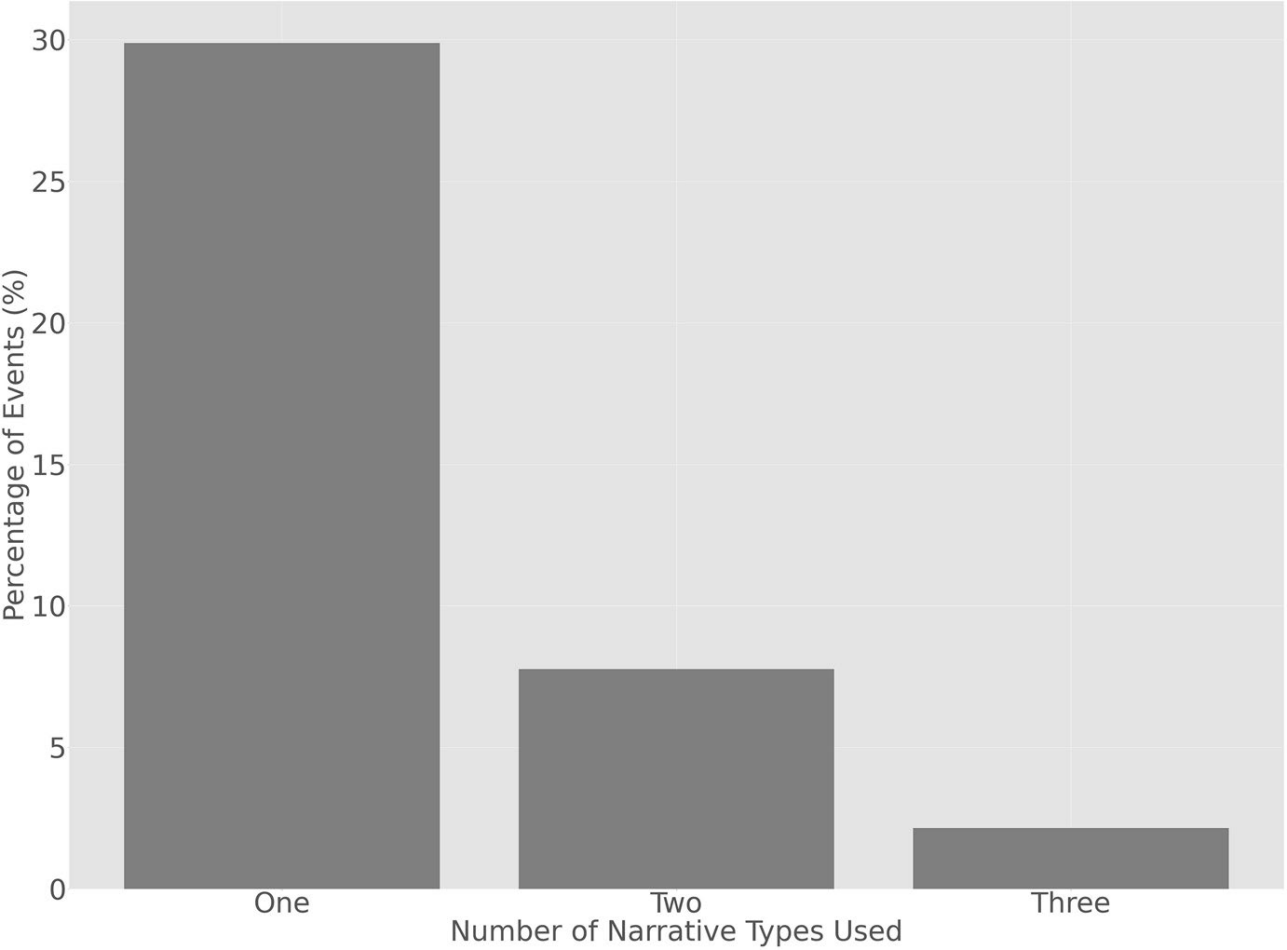
Percentage of events by number of narratives adopted

The typology of narratives—citizenship, mobilization, and confrontation—is not mutually exclusive. In fact, in a minority of protest events, citizens adopted two or more of these narratives to their strategic advantage in a single post (see Fig. 2). The co-adoption of narratives can potentially change the level of threat to the regime. For instance, a confrontational narrative is more threatening when accompanied by a mobilization narrative which calls on others to either circulate a post or join in the protest action. Conversely, a single angry, aggrieved citizen is less threatening to the state than a group of angry citizens, leading activists to disguise their collective action (Fu 2018). Moreover, an atomized individual, however, confrontational in their narrative, may arguably be easier for the authorities to silence with tried-and-tested strategies such as “emotional repression” (Hou 2020). Similarly, a citizenship narrative on its own may pose a relatively low level of threat to the state because it adopts the official rhetoric of the law in a manner reminiscent of “rightful resistance” (O'Brien and Li 2006). However, when adopted in conjunction with a mobilization narrative, the reference to laws can act in synergy with calls for collective action. Despite these co-occurrences of narratives, distinguishing the ideal types of repression narratives is useful for conceptualization purposes, even if protestors can adopt more than one narrative.

## Mention of Repression and the Adoption of Narratives

The above typology stems from a close reading of 150 randomly sampled protest events, each containing multiple microblogs that reference repression. However, not all events reference being repressed by coercive agents. In fact, 75% of the 74,415 events do not allude to repression at all, either because the protest bloggers did not experience or witness state-sanctioned coercion or because they did not dare to blog about it. By systematically comparing events that document repression with those that did not, the study assesses how particular the typology of narratives identified above is to bloggers who publicly air their grievances about being repressed.

**Fig. 3:**
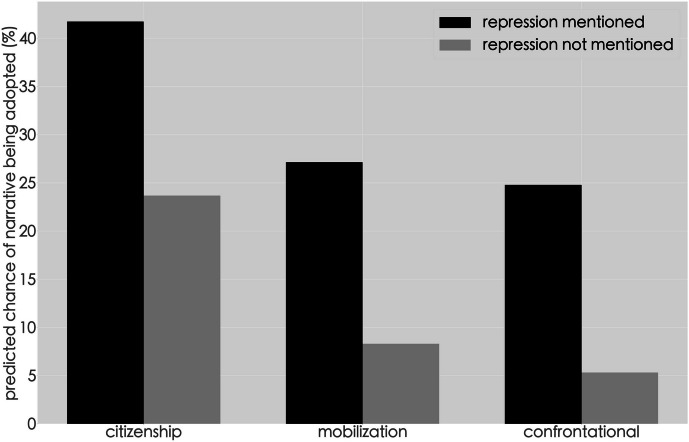
Mention of repression and adoption of narrative types. Coefficients were obtained by using the logit model to predict the likelihood of a narrative type being employed present and absent mentions of repression.

Using the methods described in the “Data and Methods” section, it finds a robust statistical relationship between mentions of repression and the adoption of terms associated with the three specific narrative types: citizenship, mobilization, and confrontational (see Figure 3). In fact, 61% of all events that reference repression adopted at least one of the three narratives in the typology, indicated by the presence of terms closely associated with each narrative. In contrast, only 32% of the events that did not mention repression adopted such narrative types. This is confirmed by the descriptive statistics which show that each of the ten most frequent terms for the narrative type appears in a higher percentage of protest events that reference repression than those that do not (see Appendix 2 Table 2). Of course, these terms are only proxies for the narrative types, as they are snippets of language that carry a specific meaning in the context of a more complicated narrative. However, there appears to be an association between the mention of repression and the way in which protestors talk about their protest experiences (Fig. 3).

First, there is a strong relationship between protest events that reference repression and a citizenship narrative (i.e., at least one of the keywords related to citizenship listed in Appendix 2 Table 2). As Fig. 3 illustrates, 42% of all events that referenced repression adopted the citizenship narrative in contrast to 24% of events that did not. A closer look at the constituent terms in Appendix 2 Table 2 reveals that “rights protection” (*weiquan*) is used most frequently, appearing in 28% of events that mention repression, in contrast to only 19% in those that did not. The other terms related to the citizenship narrative are employed more infrequently overall but are several times more likely to appear in events that mention repression. For example, the term “human rights” (*renquan*) is ten times more likely to be used in events that reference being beaten up or arrested than those that did not.

Second, there is a robust relationship between events that mention repression and at least one of the keywords associated with the mobilization narrative. Such keywords appeared in 26% of all events that mentioned repression and in only in 9% that did not. Most notably, protesters were more than four times more likely to ask other netizens to forward their post to attract attention. This significant increase is evidence that events documenting repression were significantly linked to expressions of, or calls for, mobilization in direct discursive contestation with the regime’s digital narratives.

Finally, mentions of repression were closely associated with a confrontational narrative. This narrative type is employed in 25% of events that mention repression and in only 5% that did not. Specifically, 15% of events that mention repression contain profanity, compared to 5% that did not. In other words, in events where protestors openly share how the authorities cracked down on them, bloggers were likely to be angrier in their rhetoric. While this is not surprising, their confrontational rhetoric disrupted the state’s control of the digital sphere. Challenging or berating authorities publicly upsets the state-led campaign to inject the cyber sphere with euphemistic narratives of state power and are more likely to be targeted by the authorities’ efforts to “civilize the web” (Yang 2018).

These results suggest that when protestors blogged about repression, they tended to adopt the three narrative types identified in our typology. Specifically, they were likely to speak from the positionality of a citizen rather than from that of a subject by referencing rights claims and the law. Also, they attempt to mobilize viewers by asking them to circulate their microblogs. Finally, they articulate their grievances and demands using confrontational language, including profanity and insults. This does not mean, however, that all protestors who have experienced state repression adopt one or more of these digital narratives. It may be possible that some protestors who were subjected to state-sanctioned coercion did not blog about it. Therefore, the results of this study capture the association between the narrative type and a mention of repression, rather than the experience of repression. It is also possible that the narratives are not directly related to mentions of repression, but that unmeasured variables influence both the narratives and mentions of repression. For example, it may be that in labor dispute events, bloggers are particularly prone to adopting a citizenship narrative, or that those blogging about land grabs are more likely to adopt a mobilization narrative, and that these protest types are more prone to repression than others.

To confirm that repression and the adoption of certain narrative types are indeed statistically associated, the study performed a logistic regression with mention of repression as an independent variable and each narrative type as a dependent variable. Several controls were added to test if the association between repression and narratives was robust even when other factors such as protest issue, protest location, and timing were considered. The study used the keywords provided by the Wickedonna activists to classify each event by protest issue, locality, and year and adds these variables as controls. Even with these controls, the strong association between the mentions of repression and the adoption of one or more of the narratives held (see Appendix 3 Table 3).

Still, one might reasonably assume that text length is associated with the adoption of narratives. Text length and posts per event differ markedly depending on whether repression is mentioned. In posts that do not mention repression, the average length is just 57 characters. In contrast, when protestors blog about repression, the average post length is 136 characters. In addition, there are 2.2 posts about events that do not mention repression and 4.6 posts in which repression is mentioned (see Appendix 4 Table 4). The fact that posts mentioning repression are, on average, longer is to be expected because the bloggers are very likely provoked to write more text to describe their experiences of being coerced. For example, if a blogger was beaten up and wants to highlight how this is against the law (i.e., deploying a citizenship narrative), they are likely to write a longer post about it because it simply takes more text to describe the relevant laws. Similarly, if the protest blogger wants to mobilize bystanders to join in their cause (i.e., deploying a mobilization narrative), they are likely to write a longer text as well to convince others to circulate their post. Therefore, it is to be expected that posts that describe experiences of repression are likely to be longer.

To ensure that our findings were not driven by length of text in the posts, we performed a further regression analysis that limited the sample size to posts that contained at least 300 characters. Analysis of the subsample of long posts replicated our main finding, as shown in Appendix 5 Table 5. Specifically, long posts that mention repression are more likely to adopt one or more narratives than long posts that did not mention repression. Protestors were more likely to adopt one or more of the narrative types when they blogged about repression than when they did not, even when we subset on post length (see Appendix 5 Table 5).

## Conclusion: Why Narratives of Repression Matter

This study examined how protestors and bystanders in China, a regime with high levels of state repression and censorship, blogged about their experiences of police brutality. In analyzing 74,415 protest events, it found that despite the risks, Chinese protestors did indeed blog about state repression during the early years of the Xi administration (2013–2016). In fact, one out of every four protest events (each documented by one or more microblogs) that appeared in the dataset referenced repression—being beaten and/or arrested by the police. This suggests that Chinese citizens who protested about a range of issues *routinely* blogged about state-sanctioned coercion.

The fact that protestors dared to expose police brutality in a regime like China is consequential even when it does not result in offline collective contention. In authoritarian states, the public critique of authorities tarnishes the regime’s self-image. Outraged citizens publicly criticizing local authorities and the party chips away at the regime’s public transcript of benevolent rule (Scott 1990). Autocrats across the world have invested enormously in beautifying their image and augmenting their regime’s reputation in cyberspace. Authoritarian governments such as Russia, China, and Iran have launched internet control campaigns to censor as well as seed cyberspace with pro-regime messages (King et al. 2017; Gunitsky 2015; Greitens 2013). Protestors’ digital microblogs about being beaten up by authorities tarnish the positive narrative that governments invest so much in projecting.

In particular, the Chinese party-state under Xi Jinping has gone to considerable lengths to ensure that netizens affirm and participate in the positive propaganda of the party. It has done so through a combination of policies and campaigns, including one targeting online rumors, which exemplifies the regime’s efforts to control its digital image. In an information-scarce and low-trust environment like China’s, rumors can be used by citizens as a tool in the “informational warfare” between official and unofficial discourses (Liu and Lo 2022). Therefore, the regime has an incentive to minimize online rumors. The party-state also uses artificial intelligence to recommend online content to netizens that adheres to “mainstream values.”^28^ And it has required online-content producers such as internet and media companies to promote ideological content that encourages unity and stability and to control online narratives.^29^ All of these measures suggest that for the Chinese regime, the management of its online image and of public discourse is of prime importance to the party’s rule. In this political climate, blogging about coercion disrupts the state’s public transcript about itself.

In addition to the act of blogging about repression, the study analyzed the narratives that protest bloggers used to articulate grievances and attribute blame for their problems. A close reading of microblogs documenting a random sample of 150 events found that Chinese protestors adopted three distinct narrative types in events where repression was also mentioned. First, they adopted a citizenship narrative in which they emphasized their rights as citizens who deserve to be treated with a degree of dignity. Second, they adopted a mobilization narrative by calling on bystanders to join in virtual collective action for their causes. Lastly, they also adopted a confrontational narrative, which expressed unabashed outrage at police brutality while poking fun at the regime’s propaganda about serving the people. While the citizenship closely resembles “rightful resistance” (O'Brien and Li 2006), the confrontational narrative flies in the face of the authorities, refusing to couch rights claims in legal rhetoric.

By distinguishing among three different types of narratives, this study describes a useful and meaningful variation of a discursive contention against a powerful authoritarian state that seeks to tightly control public narratives about the regime. The three narrative types are not all equal in their level of discursive challenge. The citizen narrative poses a lower level of challenge because it utilizes official discourses of legality. The confrontational narrative poses a medium level of threat because it uses expletives and other aggressive language to describe authorities’ actions, which is obviously not sanctioned. The mobilization threat perhaps poses the highest level of threat precisely because it is akin to collection action frames that directly call on bystanders to join in or to circulate the post. Each of these narrative types represents a different level of challenge to the state, one that does not necessarily translate into offline action but undoubtedly disrupts the state’s self-representation as a responsive, benevolent, and legitimate power. To the extent that the Xi Jinping administration has invested enormously in controlling online discourse via censorship, distraction, and public-opinion guidance (Repnikova and Fang 2018; Roberts 2018), these three types of online narratives of repression disrupt a powerful authoritarian leader’s best efforts at polishing his own public stature.

Together, these narratives constitute a typology of how protestors interpret and articulate state-sanctioned coercion in a politically repressive environment. As such, they belong to a broader ecosystem of digital contention and activism that have sometimes resulted in large-scale collective action. For example, digital activism was critical in the preparation phase of the Arab Spring of 2011 as protestors constructed solidarities around shared grievances and shared information about police abuse (Howard and Hussain 2013: Ch 1). Social media engagement worked in conjunction with existing civil society networks to facilitate mobilization during the 2011 Egyptian revolution (Nugent and Berman 2018). These narratives are thus generalizable to other repressive settings in which digital warriors routinely use social media to amplify their grievances and build solidarities both online and offline.

Yet, the typology of narratives identified also has elements particular to contemporary Mainland China. Specifically, the findings also suggest that the state’s efforts to control online discourse through passing laws against online rumormongering were not effective in deterring all protestors in posting about police abuse online. In fact, protestors who mention repression were three times as likely to adopt a mobilization frame than those who did not (no mention: 8.6%; mention: 26.4%). This suggests that protestors who are beaten up or arrested and who choose to expose this abuse online are also often daring enough to rally collective action despite the anti-rumor laws and related legal sanctions. In fact, the party-state’s precautions against online rumormongering may not be effective in deterring all citizens from using the cybersphere to mobilize collective action. These expressions of mobilization, even if they do not spill over onto the streets, are still troublesome to a regime that invests enormously in pumping positive energy into the cybersphere.^30^

The Chinese regime is not the only one concerned about digital exposure of its abusive power. Increasingly, unelected leaders around the world have made substantial investments in managing online information flows and online speech. For example, Russia under Putin passed a law in 2014 that required registration of popular blogs and made the bloggers liable for the accuracy of their speech.^31^ In Iran, authorities have hired an army of 42,000 volunteers to monitor online speech.^32^ In Saudi Arabia, activists who create websites critical of the government or otherwise engage in unwelcome online speech are sanctioned.^33^ Globally, internet freedoms have declined in thirty-three of sixty-five countries assessed by Freedom House.^34^ These global trends suggest that unelected rulers across the world are concerned about how their regime’s image is projected in cyberspace. Consequently, ordinary citizens blogging about authorities’ malfeasance pierces the meticulously crafted narratives of authoritarian benevolence and just rule.

## Appendix 1: Repression dictionary

Arrest: 抓走, 抓捕, 逮捕, 捉拿, 抓到, 抓进去, 抓, 捉, 押, 抓去, 抓起, 逮, 逮到, 逮住, 拘留, 擒获, 抓有, 抓并, 拘禁,羁押, 刑拘, 抓名,带进, 扣压, 刑事拘留, 软禁, 捉去,带至, 扎, 抬走, 抓住, 捕捉, 捕获, 捕, 拘捕, 阻止, 拘, 非法拘禁, 关押, 行政拘留, 关进, 抓进, 抓入, 带入, 扣押, 抓获, 扣留, 拉走, 带走, 被关在, 抓往

Arrest: to arrest, to arrest, to arrest, to arrest, to arrest, to arrest, to arrest, to arrest, to arrest, to arrest, to arrest, to arrest, to arrest criminal detention, arresting name, bringing in, withholding, criminal detention, house arrest, arresting, bringing to, stabbing, carrying away, catching, arresting, capturing, arresting, arresting, preventing, arresting, illegally detaining, imprisoning, administrative detention, shutting down, bring in

Violence: 殴打, 打人, 伤人, 动手, 暴打, 开枪, 出手, 使用暴力, 开打, 打伤, 打死, 爆打, 欧打, 打晕, 乱打, 拳打脚踢, 施暴, 大打出手, 打得, 毒打, 砍伤, 打骂, 脚踢, 痛打, 砍杀, 拳脚相加, 击打, 拳打, 大打出手, 暴力行为, 袭击, 打死, 攻击, 挨打, 揍, 打打, 弄伤, 打成, 踢, 打坏, 打断, 打了个, 群殴, 围殴, 围打, 砍死, 推倒, 踹, 打至, 弄死, 捶打, 杀, 杀人, 杀死, 打掉, 烧死, 被打

Violence: beat up, beat up, wounded, hands-on, beat up, shoot, shoot, use violence, hit, injure, beat to death, beat up, beat up, knock out, beat up, beat up, beat up, beat up, beat up, beating, slashing, beating, kicking, beating, hacking, punching, beating, punching, beating, beating, beating, beating, beating, attacking, beating, beating, beating, wounding, beat up, kicked, smashed, interrupted, beaten up, swarmed, beating, beating, hacking, knocking down, kicking, beating, slaughtering, beating, killing, killing, killing, knocking out, burning dead, beaten

## Appendix 2

**Table 2:** Usage of top ten terms associated with narrative types in the dataset

No.	Frame	Keyword	Keyword	All protest events	Events w. mention of repression	Events w/o mention of repression	% events w. mention of repression	% events w/o mention of repression
1	Citizenship	Rights protection	维权	15,840	5166	10,674	27.794	19.116
2	Citizenship	Law	法律	2355	1507	848	8.108	1.519
3	Citizenship	Urban resident	市民	1901	955	946	5.138	1.694
4	Citizenship	Rights & interests	权益	1846	988	858	5.316	1.537
5	Citizenship	Rights	权利	1606	1053	553	5.665	0.99
6	Citizenship	Citizen	公民	708	450	258	2.421	0.462
7	Citizenship	Taxpayer	纳税人	427	316	111	1.7	0.199
8	Citizenship	Human rights	人权	386	285	101	1.533	0.181
9	Citizenship	National law	国法	177	135	42	0.726	0.075
10	Citizenship	Laws & regulations	法规	161	106	55	0.57	0.098
1	Mobilization	Help forward (a post)	帮忙转	3078	1844	1234	9.572	2.237
2	Mobilization	Help spread (a post/story)	帮忙转发	2286	1361	925	7.065	1.677
3	Mobilization	Please share (a post)	求转发	2136	1339	797	6.950	1.445
4	Mobilization	Help me	帮我	1902	1087	815	5.642	1.478
5	Mobilization	Please disseminate	求扩散	1640	1010	630	5.243	1.142
6	Mobilization	Help us	帮我们	1423	782	641	4.059	1.162
7	Mobilization	Asking everyone to…	求大家	716	478	238	2.481	0.431
8	Mobilization	Help us	帮帮我们	764	443	321	2.300	0.582
9	Mobilization	Please pay attention to…	求关注	789	434	355	2.253	0.644
10	Mobilization	Save us	救我们	263	187	76	0.971	0.138
1	Confrontational	Anger	愤怒	1419	892	527	4.799	0.944
2	Confrontational	Bandits	土匪	953	797	156	4.288	0.279
3	Confrontational	Peoples’ anger	民愤	725	502	223	2.701	0.399
4	Confrontational	Damn it (vulgarity)	他妈的	490	360	130	1.937	0.233
5	Confrontational	Brute (vulgarity)	畜生	453	402	51	2.163	0.091
6	Confrontational	Wolf (vulgarity)	狼	325	200	125	1.076	0.224
7	Confrontational	Ghost (vulgarity)	鬼	233	143	90	0.769	0.161
8	Confrontational	Fucked by a dog (vulgarity)	狗日	217	165	52	0.888	0.093
9	Confrontational	Scum (vulgarity)	人渣	212	171	41	0.92	0.073
10	Confrontational	Devil (vulgarity)	鬼子	198	165	33	0.89	0.059

## Appendix 3

**Table 3:** Mention of repression and adoption of narrative types

	Citizenship			Mobilization			Confrontational		
	1	2	3	1	2	3	1	2	3
Constant	−1.17**	−1.525**	−2.187**	−2.403**	−2.802**	−2.478**	−2.879**	−2.804**	−2.84**
	−0.01	−0.025	−0.276	−0.015	−0.037	−0.265	−0.019	−0.036	−0.131
Repression	0.836**	0.869**	0.88**	1.414**	1.296**	1.293**	1.768**	1.589**	1.539**
	−0.018	−0.02	−0.02	−0.022	−0.024	−0.024	−0.025	−0.026	−0.027
Realestate		1.662**	1.621**		0.505**	0.455**		−0.006	0.109*
		−0.03	−0.031		−0.043	−0.044		−0.044	−0.046
Labor		−0.239**	−0.264**		0.3**	0.259**		−0.416**	−0.35**
		−0.029	−0.029		−0.04	−0.041		−0.042	−0.043
Fraud		0.576**	0.472**		0.361**	0.264**		0.113	0.151*
		−0.047	−0.049		−0.069	−0.07		−0.071	−0.073
Education		0.181*	0.068		0.587**	0.546**		0.572**	0.567**
		−0.064	−0.066		−0.081	−0.082		−0.079	−0.081
Medical		−0.503**	−0.476**		0.768**	0.789**		−0.052	−0.042
		−0.07	−0.071		−0.071	−0.072		−0.081	−0.082
Eviction		−0.052	−0.064		0.504**	0.536**		0.421**	0.434**
		−0.045	−0.046		−0.056	−0.057		−0.054	−0.056
Pollution		0.901**	0.814**		1.307**	1.253**		0.857**	0.804**
		−0.06	−0.062		−0.07	−0.071		−0.073	−0.075
Rural land		0.131**	0.229**		0.905**	0.912**		0.48**	0.446**
		−0.036	−0.037		−0.045	−0.046		−0.045	−0.047
Year FE			yes			yes			yes
City FE			yes			yes			yes
Obs	74424	74424	74424	74424	74424	74424	74424	74424	74424
aic	86560.12	78451.98	76555.04	54072.63	53405.41	53015.47	44495.72	43705.26	43258.03
bic	86578.55	78544.15	79486.22	54091.06	53497.58	55946.65	44514.16	43797.44	45875.81
Pseudo R2	0.025	0.116	0.145	0.068	0.079	0.097	0.102	0.118	0.138

** *p* < = 0.001, * *p* < = 0.05. Reference category: absence of repression. Cell entries display raw coefficients. Standard Errors in parenthesis

## Appendix 4

**Table 4:** Post features by presence of repression mentions

Variable	In-sample mean	Mean (Repression=0)	Mean (Repression=1)	*t*-test *p* value
Outcomes				
Citizenship narrative	0.283	0.237	0.417	0.000
Mobilization narrative	0.132	0.083	0.271	0.000
Confrontational narrative	0.104	0.053	0.248	0.000
Event issues				
Real estate	0.188	0.187	0.192	0.091
Labor	0.421	0.482	0.247	0.000
Fraud	0.040	0.042	0.031	0.000
Education	0.020	0.019	0.024	0.000
Medical	0.022	0.015	0.044	0.000
Eviction	0.054	0.042	0.089	0.000
Pollution	0.019	0.014	0.034	0.000
Rural land	0.098	0.064	0.194	0.000
Text characteristics				
Mean text length	72.600	57.339	136.000	0.000
Mean posts per event	2.428	2.224	4.553	0.000
Number of events	74,425	55,160	19,265	

Compares the means for events where repression is not mentioned and where it is. The rightmost column reports the results of a two-sided *t*-test for differences in means between the two groups assuming unequal variances

## Appendix 5

**Table 5:** Mention of repression and adoption of narrative types (long posts only)

	Citizenship			Mobilization			Confrontational		
	1	2	3	1	2	3	1	2	3
Constant	0.203**	−0.338**	−1.054**	−1.236**	−1.568**	−1.529**	−1.403**	−1.31**	−1.231**
	−0.027	−0.051	−0.126	−0.033	−0.058	−0.129	−0.034	−0.056	−0.08
Repression	0.295**	0.429**	0.469**	0.882**	0.789**	0.785**	1.064**	0.959**	0.941**
	−0.035	−0.038	−0.04	−0.039	−0.04	−0.041	−0.04	−0.041	−0.042
Realestate		1.712**	1.675**		0.222**	0.205*		−0.316**	−0.201*
		−0.064	−0.067		−0.063	−0.066		−0.062	−0.064
Labor		0.14*	0.073		0.331**	0.351**		−0.272**	−0.286**
		−0.057	−0.059		−0.064	−0.066		−0.062	−0.064
Fraud		1.302**	1.176**		0.128	0.084		0.036	0.078
		−0.11	−0.114		−0.107	−0.111		−0.102	−0.106
Education		−0.052	−0.181		0.325*	0.331*		0.264*	0.263*
		−0.097	−0.101		−0.106	−0.109		−0.102	−0.105
Medical		−0.71**	−0.664**		0.653**	0.634**		−0.184	−0.187
		−0.105	−0.109		−0.105	−0.107		−0.108	−0.11
Eviction		−0.064	−0.065		0.368**	0.407**		0.276**	0.269**
		−0.077	−0.08		−0.083	−0.085		−0.08	−0.082
Pollution		0.705**	0.703**		1.071**	1.065**		0.431**	0.417**
		−0.092	−0.096		−0.092	−0.095		−0.091	−0.094
Rural land		0.222**	0.338**		0.83**	0.828**		0.33**	0.317**
		−0.061	−0.065		−0.066	−0.069		−0.063	−0.066
Year FE			yes			yes			yes
City FE			yes			yes			yes
obs	14543	14543	14543	14543	14543	14543	14543	14543	14543
aic	19558.15	18061.32	17913.27	18165.63	17897.44	17960.05	17793.06	17581.33	17399.99
bic	19573.32	18137.17	19779.15	18180.8	17973.29	19825.93	17808.23	17657.18	18226.75
Pseudo R2	0.004	0.081	0.112	0.029	0.044	0.066	0.041	0.053	0.074

** *p* < = 0.001, * *p* < = 0.05. Reference category: absence of repression. Cell entries display raw coefficients. Standard Errors in parenthesis

## Footnotes

1 Cammett and Salti 2018 argue that ultimately, it was the dynamics of diffusion, rather than a common set of grievances, that triggered the Arab Spring of 2011.

2 On the social contract underpinning legitimation in authoritarian regimes, see Cook and Dimitrov 2017.

3 Their entries stopped on June 13, 2016, shortly before they were arrested on charges of “picking quarrels.”

4 Aggregating individual microblogs at the protest event level accounts for the dynamics of microblogging, in which users sometimes write threads or quote-tweet content from other users and thereby contribute to an unfolding debate.

5 For simplicity, we refer to these event-level collections of microblogs as “events.”

6 With respect to the agents of coercion, the three most commonly named coercive agents are the police (警察), public security (公安), and special forces (特警). It is entirely possible, however, that the protestors may mis-identify certain agents of coercion for another because they are visually difficult to distinguish (Scoggins 2023).

7 Violence: “beat up” (*ouda*); arrest: “detention” (*zhuazou*); citizenship: “protect rights” (*weiquan*); solidarity: “we” (*women*); emotion: “helpless” (*wunai*)

8 For similar approaches, see Xue et al. 2014

9 *Reuters.* 9 Sept 2013. “China Threatens Tough Punishment for Online Rumour Spreading.” https://www.reuters.com/article/us-china-internet-idUSBRE9880CQ20130909

10 Qiu, Stella and Woo, Ryan (30 August 2018). “China launches platform to stamp out ‘online rumors’”. *Reuters*. https://www.reuters.com/article/us-china-internet/china-launches-platform-to-stamp-out-online-rumors-idUSKCN1LF0HL.

11 ChinaFile.18 Sept 2013. “What is Behind China’s Big Internet Crackdown?” https://www.theatlantic.com/china/archive/2013/09/what-is-behind-chinas-big-internet-crackdown/279772/. *The Atlantic*.

12 Sun Zhigang was a migrant worker who was beaten to death by the authorities in 2003 for not having his temporary residence permit. His death at the hands of police triggered widespread online outrage, which generated public pressure leading to the abolishment of the national custody and repatriation system targeting migrant workers.

13 Zhang, Jane. 2 Sept 2021. “Weibo Bans 52 Accounts, Some with Millions of Followers, in Campaign Against Financial Misinformation on Social Media.” *South China Morning Post*. https://www.scmp.com/tech/policy/article/3147334/weibo-bans-52-accounts-some-millions-followers-campaign-against

14 *China Daily*. 22 February 2016. “State media should play due role in properly guiding public opinion.” https://www.chinadaily.com.cn/opinion/2016-02/22/content_23580181.htm.

15 State Internet Information Office. 2019. Provisions on the Governance of the Online Information Content Ecosystem. Beijing Cyber Administration of China. http://www.cac.gov.cn/2019-12/20/c_1578375159509309.htm.

16 Bandurski, David. 20 July 2015. “Taming the Flood: How China’s Leaders ‘Guide’ Public Opinion.” *ChinaFile*. Available at: https://www.chinafile.com/reporting-opinion/media/taming-flood

17 The Xi administration cracked down on this movement and the human rights lawyers who led it in 2014.

18 Local authorities often outsource violent repression to third party actors (Ong 2018, Chen 2017).

19 Event 6, Property Demolition in Shaanxi Province.

20 Event 7, Police Violence in Guangdong Province.

21 *China Daily*. 9 January 2014. “Xi: Wipe Out Judicial Corruption.” https://www.chinadaily.com.cn/kindle/2014-01/09/content_17226148.htm.

22 In selecting these keywords for the mobilization narrative, we considered the linguistic context by looking at the terms that immediately preceded and followed the keyword. For example, the most frequent term that comes after “help me” is circulate (*zhuanfa*, *zhuan* or *zhuan zhuan*). Of course, “help me” can also refer to supplication to the government, which would not qualify as a mobilization narrative. We took this into account by excluding the few posts where the keyword “help me” was mentioned in conjunction with the government.

23 Event 27, a protest against environmental pollution and police repression in 2015, Shanghai municipality.

24 Ibid.

25 Event 85, demonstration against police violence, Yulin, Guangxi Province.

26 Event 49, street vendors’ protest in 2013, Hefei, Anhui Province.

27 Event 45, forced land grab in 2015, Ningde, Fujian Province.

28 Gan, Nectar. 11 September 2019. “China’s internet regulator orders online AI algorithms to promote ‘mainstream values.’” *South China Morning Post*. https://www.scmp.com/news/china/politics/article/3026784/chinas-internet-regulator-orders-online-ai-algorithms-promote

29 Zhong, Raymond, Paul Mozur, Aaron Krolik and Jeff Kao. “Leaked Documents Show How China’s Army of Paid Internet Trolls Helped Censor the Coronavirus.” *ProPublica*. https://www.propublica.org/article/leaked-documents-show-how-chinas-army-of-paid-internet-trolls-helped-censor-the-coronavirus

30 Bandurski, David. Dec. 15 2015. “The CCP’s ‘Positive Energy’ Obsession.” *China Media Project*. https://chinamediaproject.org/2015/12/15/chinas-obsession-with-positive-energy/

31 Freedom on the Net: The Rise of Digital Authoritarianism. 2018. *Freedom House*. https://freedomhouse.org/report/freedom-net/2018/rise-digital-authoritarianism

32 Freedom on the Net: The Crisis of Social Media. 2019. *Freedom House*. https://freedomhouse.org/sites/default/files/2019-11/11042019_Report_FH_FOTN_2019_final_Public_Download.pdf

33 “Freedom on the Net 2019: Saudi Arabia.” 2019. *Freedom House*. https://freedomhouse.org/country/saudi-arabia/freedom-net/2019

34 Freedom on the Net: The Crisis of Social Media. 2019. *Freedom House*. https://freedomhouse.org/sites/default/files/2019-11/11042019_Report_FH_FOTN_2019_final_Public_Download.pdf
